# *Withania coagulans* fruit extract antiulcerogenic effect: comparative study with lansoprazole and ranitidine in rats

**DOI:** 10.3389/fmed.2025.1544422

**Published:** 2025-06-25

**Authors:** Naheed Amir, Hassan Abu Damir, Karam Ghazal-Aswad, Mohamed Hassan, Mukhtar Adem, Mahmoud A. Ali, Salim Bastaki, Ernest Adeghate, Abdu Adem

**Affiliations:** ^1^Department of Biological and Biomedical Sciences, Medical College, The Aga Khan University, Karachi, Pakistan; ^2^Department of Medical Sciences, College of Medicine and Health Sciences, Khalifa University, Abu Dhabi, United Arab Emirates; ^3^Division of Hospital Medicine, University of North Carolina, Chapel Hill, NC, United States; ^4^Department of Pharmacology & Anatomy, College of Medicine & Health Sciences, United Arab Emirates University, Al Ain, United Arab Emirates

**Keywords:** *Withania coagulans*, Wister rats, gastric ulcer protection, histamine blocker, anti-oxidative stress, anti-inflammatory, lansoprazole, ranitidine

## Abstract

**Background:**

Peptic ulcer disease (PUD) arises from an imbalance between harmful factors like gastric acid and pepsin, and the protective mechanisms of the gastrointestinal lining, particularly the mucus–bicarbonate barrier. Standard treatments include proton pump inhibitors (e.g., lansoprazole) and histamine H₂-receptor antagonists (e.g., ranitidine), but these can have adverse effects. *Withania coagulans*, a plant used in Ayurvedic medicine, has traditionally been considered to have anti-ulcer properties. This study investigated the potential of *W. coagulans* fruit extract to protect against gastric ulcers, possibly via H₂ receptor antagonism.

**Aim:**

To evaluate the gastroprotective effects and underlying mechanisms of *W. coagulans* fruit extract in a rat model of gastric ulcer.

**Methods:**

A dose–response study was conducted using rats divided into six groups: naïve, ulcer control, and four groups treated with *W. coagulans* extract (1, 5, 10, or 20 mg/kg). Acidified ethanol was used to induce ulcers. In another experiment, pylorus-ligated rats were used to assess the extract’s effect on gastric acid secretion in response to dimaprit, a histamine analog. For efficacy comparison, rats were pretreated with *W. coagulans*, lansoprazole, or ranitidine before ulcer induction. Gastric tissues were analyzed for biochemical markers, including cytokines, mucus, prostaglandin E2 (PGE2), and myeloperoxidase activity.

**Results:**

The 10 mg/kg dose was most effective in reducing gastric ulceration. The extract reduced gastric acid secretion, like H₂ blockers. It also showed stronger antioxidant activity in gastric tissues compared to lansoprazole and ranitidine. Additionally, it reduced pro-inflammatory cytokines (IL-1*β*, TNF-*α*, IL-6), increased anti-inflammatory cytokines (IL-10, TGF-β), enhanced mucus and PGE2 production, and lowered myeloperoxidase activity.

**Conclusion:**

*Withania coagulans* fruit extract at 10 mg/kg significantly protects against acid-induced gastric ulcers. Its effects are comparable to H₂ receptor blockers and include notable antioxidant and anti-inflammatory benefits.

## Introduction

Peptic Ulcer Disease (PUD) is global and caused by many factors that favor gastric acid and pepsin production to disrupt the mucus/bicarbonate barrier of gastric/duodenal mucosae (1, 2). The disease is mostly attributed to *H. pylori* infection, nonsteroidal anti-inflammatory drugs (NSAID), Zollinger-Elisson syndrome and recently Aldronate (3–5) while old age, genetic factors and hypercalcemic status are common risk factors (6, 7). Lifestyle such as smoking, alcoholism, certain types of food and stress are predisposing factors exacerbating the condition (3, 8). The hormone Gastrin stimulates enterochromaffin- like cells (ECL) to release histamine which in turn stimulates HCL production (9). Neurotransmitters, acetylcholine and gastrin related peptide mainly from vagal and enteric neurons are also involved in gastric acid stimulation (10). On the other hand, somatostatin predominantly secreted by fundic antral Delta cells (D cells) inhibits acid secretion via activation of somatostatin receptor 2 (SSTR2) receptors in parietal and ECL cells (11, 12). In addition, a number of neuropeptides modulate gastric secretion either by stimulation or inhibition of gastric acid production (13, 14).

Acute or chronic peptic ulcer disease (PUD) poses a significant threat to health, as it is often associated with varying degrees of inflammation and potentially severe complications (3, 15). The formation of ulcers involves disruption of the epithelial lining, leading to necrosis, delayed healing, pain, impaired blood circulation, risk of infection, and elevated levels of pro-inflammatory cytokines (16). The inflammatory response involves the activation of neutrophils, helper T cells (Th), macrophages, and other granulocytes, which release cytokines such as TNF-*α*, IL-1, IFN-*β*, and IL-6—further amplifying inflammation and pain (17).

In response to tissue injury, anti-inflammatory cytokines including IL-4, IL-10, and TGF-β are also secreted—mainly by macrophages—to promote tissue repair and resolution of inflammation. Among pro-inflammatory markers, IL-1β, TNF-*α*, and IL-6 play central roles in the pathogenesis of gastric ulcers. IL-1β is elevated in conditions such as ulcer formation, cellular injury, tumors, and immune disorders, intensifying inflammatory processes (18, 19). TNF-*α* contributes to acute inflammation, prolonged bleeding, and endothelial dysfunction (20). It also initiates complex signaling cascades that may lead to apoptosis, activate NF-κB, or stimulate the MAPK pathway, further enhancing cytokine production and cellular stress (21–23).

IL-6 performs multiple functions, including promoting acute inflammation, inducing acute-phase protein and platelet production, and altering hematopoiesis in the bone marrow. It can also exert anti-inflammatory effects under certain conditions (24–27). IL-6 additionally attracts neutrophils and monocytes/macrophages to the ulcer site, reinforcing the inflammatory response.

On the other hand, IL-4 stimulates transcription factors such as PPAR-*γ* and GATA3, encouraging the differentiation of naïve CD4 + T cells into Th2 cells that support tissue repair (28). Overall, anti-inflammatory cytokines such as IL-4, IL-10, and TGF-*β* are crucial for counterbalancing inflammation and promoting healing (29, 30). Under normal physiological conditions, a delicate balance between pro- and anti-inflammatory cytokines is maintained to ensure homeostasis and tissue recovery. Standard drug regimen used for ulcer treatment include proton pump inhibitors (H+/K + ATPase) as Lansoprazole or histamine-2 (H2) blockers as Ranitidine are with varying efficacy (31, 32). However, side effects such as hypergastrinemia, hepatic dysfunction, skin hypersensitivity, headache, gastric problems, bleeding, joint and muscle pain, anxiety/depression, etc. may be observed (33, 34). Genotoxic and carcinogenic effects have been evaluated among a number of gastrointestinal drugs including those used for PUD and variable adverse effects were reported (35). In recent years, the FDA identified low levels of N-nitrosodimethylamine (NDMA), a known carcinogen, in certain ranitidine products. In response, the agency issued alerts to patients and healthcare professionals regarding its findings, leading to the temporary withdrawal of the drug from some healthcare facilities (36).

Medicinal plants have been used extensively in folk medicine and contributed effectively to the discoveries of new drugs (37). Assessment trials of medicinal plants for the treatment of peptic ulcer have been an active field where some essential oil components such as limonene; sesamol; *α*-pinene; (−) myrtenol among others proved to be potentially effective (38–41). Based on biological properties, *Withania coagulans* Dunal, family Solanaceae, is among the herbs alleged to have some ulcer curative effects (42). *Withania coagulans* has been widely used in Ayurveda medicine in Indian subcontinent and some South, and East Asian countries (43, 44). It exhibits antidiabetic, antioxidant, anti-inflammatory, antihypertensive, immunosuppressive, antimicrobial, antimutagenic, analgesic and many other properties along with wound healing benefits (45–47) and thus we speculated that it may exhibit an antiulcerogenic effect. Studies have already demonstrated that extracts from the fruits and seeds of *W. coagulans* exhibit significant free radical scavenging activity, which has been attributed to their high phenolic and flavonoid contents. (48, 49). Other, phytochemical constituents of the plant have also been isolated from the whole plant, leaves, fruits, seeds, bark and root. These include biologically active molecules such as steroidal lactones as withaferins, withanolides, alkaloids, amino and organic acids, saponins, tannins, fatty acids beside many minor constituents which induce curative effects (50, 51). *Withania coagulans* fruits are safely used in the preparation of cottage cheese in several Asian countries. The plant was not reported to induce any toxicity to albino rats at a dose rate of 2000 mg/kg causing no histopathological lesions in liver, heart, kidney and pancreas however, a slight increase in SGOT, SGPT and urea levels were observed at that high dose (47). We hypothesized that *W. coagulans* fruit extract induces antiulcerogenic effects by acting as a histamine-2 blocker. Therefore, the aim of this study was twofold: firstly, to assess the protective effects of *W. coagulans* fruit extract against ethanol-induced gastric ulcer in rats compared to standard PUD drugs, Lansoprazole and Ranitidine. Secondly, to investigate the possible mechanisms through which the extract exerts its effects. This possibility was addressed by investigating the effects of *W. coagulans* fruit extract on gastric acid secretion and somatostatin production in rats after pylorus ligation with or without administration of exogenous histamine (Dimaprit).

## Materials and methods

### Chemicals

Bovine serum albumin, 5-sulfosalicylic Acid (SSA), naphthalene diamine dihydrochloride, sulphanilamide, phosphoric acid, 3,3,5,5′-Tetramethylbenzidine (TMB), glutathione (GSH) assay kit and all other required chemicals, if not specified were purchased from Sigma-Aldrich (Sigma Chemical Co., St. Louis, MO, United States). All chemicals used in the present study were of analytical grade. Malondialdehyde (MDA) assay kit was purchased from Northwest Life Science Specialties (WA, United States). Cytokines duo set ELISA kits were purchased from R&D Systems (Minneapolis, MN, United States). EnzChek® myeloperoxidase (MPO) activity assay kit was purchased from Life Technologies (NY, United States).

### Animals and diet

Male Wister rats (230 to 250 g) bred in the animal research facility of the College of Medicine and Health Sciences, United Arab Emirates University, Al Ain, UAE were used. The animals were housed under standard laboratory conditions; (22 ± 2°C and 65 ± 5% humidity) and maintained on a 12-h light/dark cycle. The animals had free access to food and water and were fed on a commercially available standard rat diet. A maximum of four rats were housed per cage and acclimatized to the laboratory conditions prior to the commencement of the experiment.

The experimental protocols were approved by the Institutional Animal Ethics Committee of College of Medicine and Health Sciences (IAEC CMHS), United Arab Emirates University, Al Ain, UAE, and conducted according to the criteria outlined in the guide for the care and use of laboratory animals by the National Academy of Sciences and Animal Research: Reporting of *In Vivo* Experiments (ARRIVE) list of guidelines.

### Preparation of the *Withania coagulans* aqueous fruit extract

The complete identification and authentication for *W. coagulans* plants as well as fruits (Verification number: KUH-99864) was done by Dr. Muneeba Khan, a plant taxonomist from the Center for plant Conservation- Herbarium and Botanic Garden, University of Karachi, Pakistan.

The extraction of *W. coagulans* was carried out as previously described by Ojha et al. (46). Briefly, after removing the calyx, the fruits of *W. coagulans* (0.28 g/100 mL) were soaked in distilled water overnight. The softened fruits were then mechanically dispersed using a sterile cottonwood applicator (Hardwood Products Company, Guilford, United States). The resulting mixture was filtered through cheesecloth to obtain the aqueous extract. No further processing was performed, and the extract was used directly for oral gavage. Dosage was calculated based on the concentration of this suspension.

### Dose optimization study using ethanol-induced gastric ulcer model

To establish the effective dose of *W. coagulans*, a dose–response study was conducted. After an overnight fast, rats were randomly divided into six groups (*n* = 6–8): naive (no treatment), ulcer control (vehicle), and *W. coagulans*-treated groups receiving 1, 5, 10, or 20 mg/kg. Thirty minutes post-treatment, gastric ulcers were induced by oral administration of 1 mL of acidified ethanol (60% ethanol in 150 mM HCl). One hour later, rats were sacrificed by decapitation, and their stomachs were excised, opened along the greater curvature, rinsed with saline, and examined for macroscopic lesions. The ulcer index was calculated based on the length and severity of lesions. Gastric tissues were fixed in 10% formalin, embedded in paraffin, sectioned, and stained with hematoxylin and eosin for histological evaluation.

### Comparative gastroprotective study with standard drugs

To compare the protective efficacy of *W. coagulans* with standard anti-ulcer drugs, overnight fasted rats were allocated into five groups: untreated ulcer control, D. H₂O, *W. coagulans* (10 mg/kg, p.o.), lansoprazole (10 mg/kg, p.o.), and ranitidine (20 mg/kg, p.o.). Thirty minutes after treatment, all animals except the naive group received 1 mL of acidified ethanol (60% ethanol in 150 mM HCl) via oral gavage to induce gastric injury. One hour post-induction, rats were sacrificed by decapitation. The abdomen was incised, and the stomachs removed, cut open along the greater curvature, and rinsed with saline to remove any adherent particles. The open stomach was spread on a sheet of cork to have a clear view of gastric lesions in the gastric mucosa. The total lengths of hemorrhagic lesions, which were approximately 1 mm in length and formed in the glandular portion of the gastric mucosa, were taken as ulcer index. An observer who was unaware of the drug treatments confirmed the ulcer index. The percentage reduction of the ulcer index in the drug-treated groups was calculated from the saline-treated groups. Stomach tissues were snap frozen in liquid nitrogen until tissue homogenate preparation for different measurements. Tissues were fixed and stained for histological analysis. The use of 60% ethanol in 150 mM HCl to produce an ulcerogenic effect was based on earlier observation that over 50% ethanol provided a reproducible model of gastric damage (52, 53, 91).

### Assessment of gastric acid secretion and somatostatin levels in pyloric ligation model

To assess the effects of *W. coagulans* on gastric acid secretion and somatostatin levels, a pyloric ligation model was used. Rats were assigned to four groups (*n* = 6–8): (1) control (distilled water, p.o.), (2) *W. coagulans* extract (10 mg/kg, p.o.), (3) dimaprit (10 mg/kg, i.p.) plus distilled water, and (4) dimaprit plus *W. coagulans* extract. All treatments were administered 30 min before surgical pyloric ligation under anesthesia. Four hours post-ligation, the animals were sacrificed, and gastric contents were collected by gently expressing the stomach contents into pre-weighed tubes. Gastric juice volume was measured, and total acidity was determined by titration with 0.01 N NaOH using phenolphthalein as an indicator. Acid output was calculated and expressed as mmol/4 h. Samples were stored at −40°C for subsequent somatostatin quantification by ELISA.

#### Preparation of tissue homogenate

Gastric glandular mucosa tissue was washed with ice-cold phosphate buffer saline (PBS) and scrapped. The tissue was weighed and homogenized with 10 volumes of ice-cold high KCl lysis buffer (10 mM Tris–HCl, pH 8.0, 140 mM NaCl, 300 mM KCl, 1 mM EDTA, 0.5% Triton X-100 and 0.5% sodium deoxycholate) with complete protease inhibitor cocktail through 2.8 mm ceramic beads by using bead ruptor 4 homogenizers (Omni international, United States). The resulting homogenates, after 30 min incubation on ice, were centrifuged at 15000 rpm for 30 min at 4°C. The resulting supernatant was stored at −40°C until the ELISA analysis.

#### Measurement of inflammatory cytokines

##### IL1-*β*, IL-6, TNF-*α* and IL-10

Enzyme immunoassay of IL1-β, IL-6, IL-10 and TNF-α in gastric homogenate was performed by using commercial rat Duo set sandwich ELISA. In brief, the wells of a 96 micro titer plate were coated with primary antibody in phosphate buffer saline (PBS), (100 μL/well) for overnight at room temperature, washed with phosphate-buffered saline containing 0.05% Tween (PBST), and then blocked with albumin bovine serum in PBS for 1 h. After washing, plates were incubated with gastric tissue homogenates and respective standards for 2 h. After washing with PBST, a detection antibody was added for 2 h. 100 μL of horse reddish peroxidase (HRP) was added for half an hour, after the washing. The substrate, TMB-ELISA was added, and the color intensity read at 450 nm with a microplate reader (Tecan Group Ltd., Männedorf, Switzerland). Cytokines levels were expressed as picogram per milligram of protein.

#### Measurement of oxidative stress markers

##### MDA assay

The level of MDA in the homogenate, prepared by cold high KCL lysis buffer from each group was measured by using the Malondialdehyde Assay kit (Northwest Life Science Specialties, LLC 16420 SE McGillivray, Suite 103, PMB 106, Vancouver, WA 98683, United States). The assay is based on the reaction of MDA with thiobarbituric acid (TBA); forming a MDA-TBA2 adduct that absorbs strongly at 532 nm. In brief, 250 μL tissue homogenate was added to 250 μL of 1 M phosphoric acid and 250 μL of butylated hydroxytoluene in ethanol, and then the mixture was heated at 60°C for 60 min. The suspension was cooled to room temperature, centrifuged at 10,000 rpm for 2–3 min and the pink colored supernatant was taken for spectroscopic measurements at 532 nm for the assay of MDA. MDA concentrations were expressed in μM.

#### Superoxide dismutase

Superoxide dismutase (SOD) assay was performed according to the manufacturer’s protocol. This colorimetric assay utilized the tetrazolium salt for detection of superoxide radicals generated by xanthine oxidase and hypoxanthine. One unit of SOD enzyme was required to inhibit 50% dismutation of the superoxide radical. The inhibition of color development was measured at 450 nm by using Emax Plus microplate reader (Molecular devices, CA 94089, United States). Results were expressed in units per 100 mg of gastric mucosa.

#### Catalase activity

Catalase activity (CAT) levels were measured by Catalase Assay Kit which utilizes the peroxidatic function of CAT for determination of enzyme activity. The method was based on the reaction of the enzyme with methanol in the presence of an optimal concentration of H_2_O_2_. The formaldehyde produced was measured spectrophotometrically at 540 nm, with 4-amino-3-hydrazino- 5-mercapto-1, 2, 4-trizazole as the chromogen by using Emax Plus microplate reader (Molecular devices, CA 94089, United States). CAT activity in tissue homogenates were expressed as nmol/min/100 mg gastric mucosa.

### GSH assay

#### Sample preparation for GSH ASSAY

Tissue homogenates were deproteinized by mixing 150 μL of supernatant from prepared homogenates with 150 μL of 5% (w/v) sulfosalicylic acid (SSA) solution and vortexed immediately followed by 10 min incubation on ice. Samples were centrifuged at 10,000 rpm for 10 min to remove the precipitated protein. The resulting supernatant was removed and stored at − 80°C until the GSH assays was carried out.

### Reduced glutathione

GSH content in stomach tissue homogenate was estimated according to the method described by the suppliers of the assay kit (Sigma –Aldrich 3,050 Spruce St. Louis, MO 63103). The measurement of GSH uses a kinetic assay in which catalytic amounts (nmoles) of GSH caused a continuous reduction of 5, 5¢- dithiobis (2-nitrobenzoic acid; DTNB) to TNB and the glutathione disulfide (GSSG) formed was recycled by glutathione reductase and NADPH. The yellow color product, 5-thio-2-nitrobenzoic acid (TNB) was measured spectrophotometrically at 412 nm within 5 min of 5, 5-dithio-bis (2-nitrobenzoic acid) addition, against a blank with no homogenate. GSH concentration was expressed as μM.

### Determination of myeloperoxidase

The MPO was measured by sandwich ELISA according to manufacturer’s protocols. Briefly, 100 μL of the sample and standards were added to the 96 well microtiter plate, coated with antibodies recognizing rat MPO, for 1 h at room temperature. After washing, 100 μL /well biotinylated trace antibody was added for 1 h at room temperature. After the washing, Streptavidin-peroxidase conjugate was added to bind with biotinylated trace antibody for 1 h at room temperature. The TMB-ELISA substrate was added for 30 min at room temperature, after washing the plate. The enzyme reaction was stopped by oxalic acid. The absorbance was read at 450 nm, after adding stop solution, with a microplate reader (Tecan Group Ltd., Männedorf, Switzerland). MPO levels were expressed as ng per milligram of protein.

### Determination of nitric oxide

Nitric Oxide level was estimated by measuring the concentration of nitrate and nitrite in the gastric mucosa homogenate using Cayman colorimetric assay kit. Briefly, nitrate was enzymatically reduced to nitrite by adding nitrate reductase. After a 3 h incubation at room temperature, nitrite was converted to purple azo dye by adding Griess I and Griess II (1:1), followed by 10-min incubation at room temperature. Nitrite levels were then determined spectrophotometrically at an absorbance of 540 nm using Emax Plus microplate reader (Molecular devices, CA 94089, United States). NO was expressed as μM per milligram of tissue.

#### Determination of transforming growth factor-*β*

The Transforming growth factor (TGF-β) was measured by MyBiosource sandwich ELISA kit according to manufacturer’s protocols. Briefly, sample and standards were added to the coated 96 well micro titer plate for 2 h. at 37°C. After removal of samples, detection reagent A was added for 1 h. at 37°C. After washing, detection reagent B was added for 1 h. at 37°C. The TMB-ELISA substrate was added, after the washing. The absorbance was read at 450 nm, after adding stop solution, with an Emax Plus microplate reader (Molecular devices, CA 94089, United States). TGF-*β* levels were expressed as picogram per milligram of gastric tissue.

### Assay of prostaglandin E2 in gastric mucosa

Enzyme immunoassay of PGE2 in gastric mucosa and serum was performed by using commercial kit (Cayman Chemical Company, Ann Arbor, MI, United States). Briefly, after dissection, stomachs were washed with ice-cold PBS, and the gastric mucosa were rapidly scraped from the underlying tissue layers of gastric on ice. The mucosa was weighed, minced by forceps, and homogenized with 3 volumes of cold phosphate buffer (PBS 0.1 mol/L, pH 7.4, containing 1 mM EDTA and 10 μM indomethacin) per gram of tissue using 2.8 mm ceramic beads by using bead ruptor 4 homogenizer (Omni international, United States). The color intensity was read at 450 nm with an Emax Plus microplate reader (Molecular devices, CA 94089, United States). PGE2 concentration was expressed as picogram per gram of gastric tissue.

### Determination of gastric wall mucus

Gastric wall mucus was determined according to the modified procedure of Carne et al. (54). The glandular segment of the stomach was separated from the lumen of the stomach, weighed (100 mg), and transferred immediately to 1 mL of 0.1% w/v Alcian blue solution (in 0.16 M sucrose solution buffered with 0.05 M sodium acetate at pH 5.8). Tissue was stained for 2 h in Alcian blue, and excess dye was removed by two successive rinses with 1 mL of 0.25 M sucrose, first for 15 min. and then for 45 min. Dye complexed with the gastric wall mucus was extracted with 500 μL of 0.5 M magnesium chloride which was intermittently shaken for 1 min at 30-min intervals for 2 h. The resulting emulsion was centrifuged at 4000 rpm for 10 min and the absorbance of aqueous layer was recorded at 580 nm. The quantity of Alcian blue extracted per gram of wet glandular tissue was then calculated.

### Assay of somatostatin in gastric juice

Competitive Enzyme immunoassay of Somatostatin in gastric juice was performed according to manufacturer’s protocol. Briefly, the immunoplate was pre-coated with secondary antibody and the nonspecific binding sites were blocked. The secondary antibody was allowed to bind to the Fc fragment of the primary antibody whose Fab fragment was competitively bound by both biotinylated peptide and peptide standard or targeted peptide in samples during 2 h’ incubation at room temperature (20–23°C) on orbital shaker at 300–400 rpm. The interaction of biotinylated peptide with streptavidin-horseradish peroxidase (SA-HRP) was catalyzed by the substrate solution. The intensity of the color is directly proportional to the amount of biotinylated peptide-SA-HRP complex but inversely proportional to the amount of the peptide in standard solutions or samples. The color intensity was read at 450 nm with an Emax Plus microplate reader (Molecular devices, CA 94089, United States). Somatostatin concentration was expressed as nanogram per ml of gastric juice.

#### Histopathological methods

After macroscopic observation, samples of the stomach were subsequently excised for microscopic observation. The tissues were fixed immediately in 10% buffered formalin, processed in tissue processor (ATP1-220, Triangle Biomedical Sciences, INC., Durham, United States), embedded in paraffin wax, sectioned into 5 μM thick in microtome (RMT 202A, Bioevopeak Co., Ltd. Jinan, Shandong, China), deparaffinized with xylene, stained with hematoxylin–eosin (H&E; Thermo Fisher Scientific; United Kingdom) as per (55) and viewed under the light microscope (Olympus digital microscope U-APT, Olympus, Tokyo, Japan).

### Statistical analysis

Statistical analysis of data was carried out using IBM SPSS 24.0 software. One-way ANOVA test was used to determine the significance of means between the different groups. The data were expressed as mean±SEM. Values of *p* < 0.05 were considered significant.

## Results

### Protective effects of *Withania coagulans* against gastric ulcers (antiulcer activity)

#### Ulcer index of *Withania Coagulans* fruit extract

**Figure 1:**
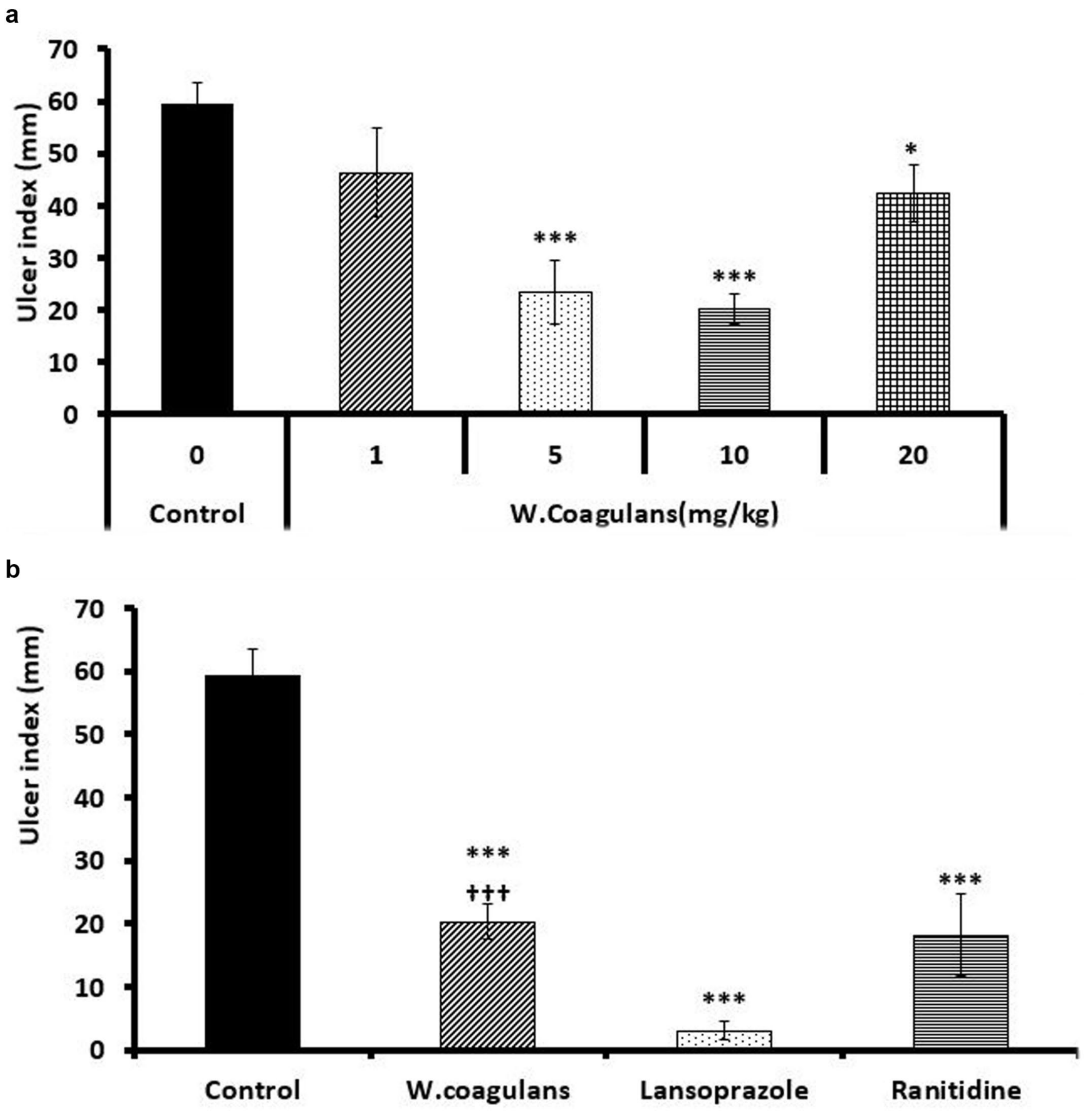
**(a)** Ulcer index (mm) in the gastric mucosa of rats pretreated with 0 (control), 1, 5, 10 and 20 mg/kg *Withania coagulans* fruit extract then challenged with acidified ethanol. *denotes significance from control. Increasing number of signs denote increasing levels of sig. (*p* < 0.05; *p* < 0.001). **(b)** Ulcer index (mm) in the gastric mucosa of rats pretreated with 10 mg/kg *W. coagulans* fruit extract compared to Control, Lansoprazole and Ranitidine. Signs denote: *Sig from Control; †Sig. from Lansoprazole; *p* < 0.001.

Figure 1a shows the protective effects of different pretreatment doses of *W. coagulans* fruit extract (0, 1, 5, 10, 20 mg/Kg) against gastric ulcers induced by acidified ethanol in rats. The dose response curve clearly indicated that pretreatment of rats with 10 mg/kg *Withania* fruit extract was the most effective dose causing the least ulcer index measurements compared to control (*p* < 0.001). However, 1 and 20 mg/kg. *Withania* fruit extract were not so effective against alcohol-induced gastric ulcers. 5 mg/kg dose of extract encountered a significant protective effect as 10 mg/kg fruit extract but with wider data dispersion.

#### Ulcer index of *Withania Coagulans* fruit extract compared to lansoprazole and ranitidine

Figure 1b shows ulcer index of *W. coagulans* fruit extract (10 mg/kg) compared to Lansoprazole (10 mg/kg) and Ranitidine (20 mg/kg) doses administered as pretreatment to protect against gastric ulcers induced by acidified ethanol to rats. *Withania* was equally effective as Ranitidine in reducing gastric ulcer index compared to Control (*p* < 0.001) however, Lansoprazole displayed a better effect (*p* < 0.001) compared to *Withania*.

### *in-vivo* gastric acid secretion

**Figure 2:**
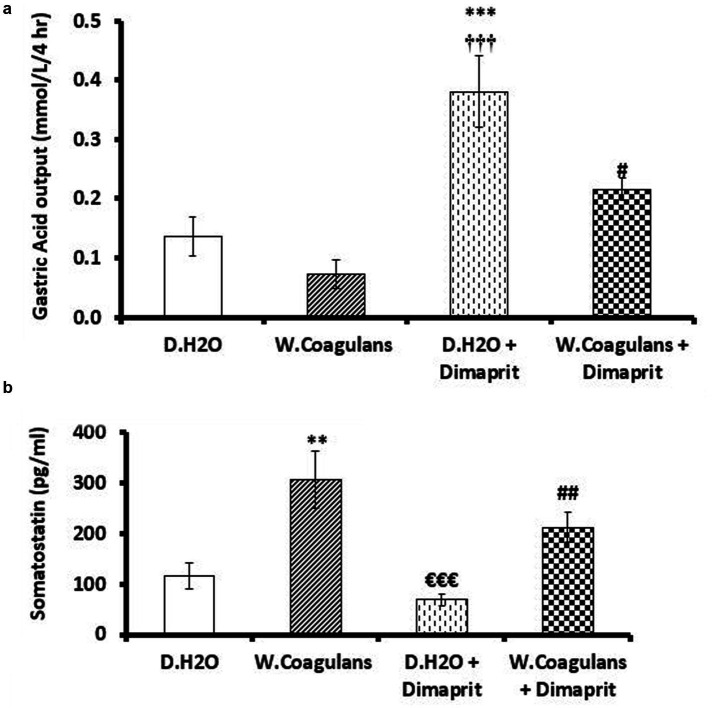
**(a)** Gastric acid output (nmol/L/4hr) in gastric juice of rats after pylorus ligation and then administration of either of: 10 mg/kg *W. coagulans* fruit extract; distilled water (DH: O) + Dimaprit; *W. coagulans* fruit extract+ Dimaprit or DHO only (control). Signs denote: *Sig. from DH: O; #Sig.from Dimaprit; †Sig from *W. coagulans*. Increasing number of signs denote increasing levels of sig. (*p* < 0.05; *p* < 0.001). **(b)** Somatostatin contents (ng/ml) in gastric juice of rats after pylorus ligation and then administration either of: 10 mg/kg *W. coagulans* fruit extract; distilled water (DH: O) + Dimaprit; *W. coagulans* fruit extract+ Dimaprit or DHO only (control). Signs denote: *Sig. from D H2O (*p* < 0.01); #Sig. from D. H2O + Dimaprit (*p* < 0.01); €Sig from *W. coagulans*. Increasing number of signs denote increasing levels of sig. (*p* < 0.01; *p* < 0.001).

Gastric acid output in pylorus-ligated rats receiving different treatment doses via gastric gavage is shown in Figure 2a. Administration of Dimaprit with distilled water (Dimaprit + D. H₂O) significantly increased gastric acid output compared to both the control group (D. H₂O alone) and the group treated with *W. coagulans* fruit extract alone (*p* < 0.001). Co-administration of *W. coagulans* extract with Dimaprit significantly reduced gastric acid output compared to the Dimaprit + D. H₂O group (*p* < 0.05). However, gastric acid levels in the combination group remained higher than those in rats treated with the fruit extract alone.

As shown in Figure 2b, somatostatin levels in the gastric contents were significantly higher in rats treated with *W. coagulans* fruit extract compared to the H₂O + Dimaprit group (*p* < 0.001) and the H₂O control group (*p* < 0.01). Additionally, the *W. coagulans* + Dimaprit group exhibited significantly elevated somatostatin levels compared to the H₂O + Dimaprit group (*p* < 0.01).

### Gastric wall mucus

**Figure 3:**
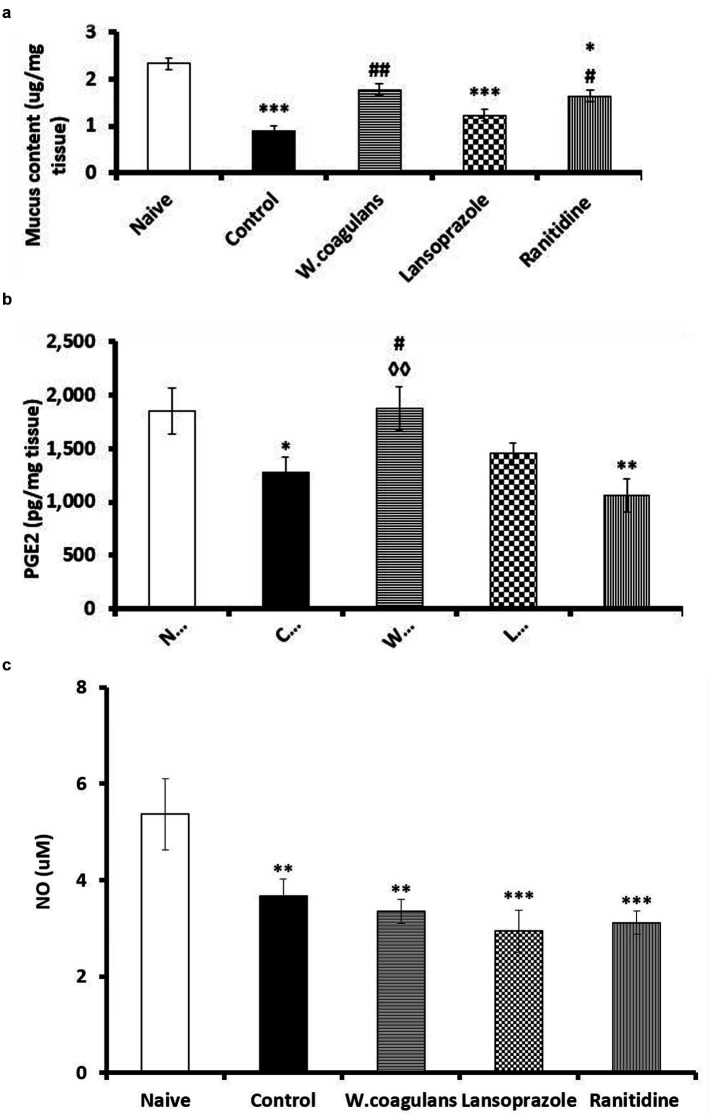
**(a)** Mucus content (μg/mg tissue) in the gastric mucosa of rats pretreated either with *W. coagulans* fruit extract (10 mg/kg); Lansoprazole (10 mg/kg); Ranitidine (20 mg/kg) or left as positive Control which were then challenged with acidified ethanol per os. The Naïve group was left untreated. Signs denote: *Sig. from Naïve; #Sig. from Control. Increasing number of signs denote level of sig. (*p* < 0.05; *p* < 0.01; *p* < 0.001). **(b)** PGE2 (pg/mg tissue) in the gastric mucosa of rats pretreated either with *W. coagulans* fruit extract (10 mg/kg); Lansoprazole (10 mg/kg); Ranitidine (20 mg/kg) or left as positive Control which were then challenged with acidified ethanol per os. The Naïve group was left untreated. Signs denote: *Sig. from Naïve; # Sig. from Control; †Sig. from Lansoprazole; ◇Sig. from Ranitidine. Increasing number of signs denote level of sig. (*p* < 0.05; *p* < 0.01). **(C)** ΝΟ (μM) in the gastric mucosa of rats pretreated either with *W. coagulans* fruit extract (10 mg/kg); Lansoprazole (10 mg/kg); Ranitidine (20 mg/kg) or left as positive Control which were then challenged with acidified ethanol per os. The Naïve group was left untreated. Signs denote: *Sig. from Naïve. Increasing number of signs denote level of sig. (*p* < 0.01; *p* < 0.001).

The results of gastric wall mucus content in the Withania coagulans, lansoprazole, ranitidine, control, and naïve groups of rats are shown in Figure 3a. The control group exhibited significantly lower gastric mucus levels compared to the naïve (*p* < 0.001), *W. coagulans* (*p* < 0.01), and ranitidine (*p* < 0.05) groups. Mucus content in the *W. coagulans* group was comparable to that of the naïve group. However, both the lansoprazole (*p* < 0.001) and ranitidine (*p* < 0.05) groups showed significantly reduced mucus levels compared to the naïve group.

### Gastric prostaglandins E2

The PGE2 levels in the gastric mucosal homogenate of experimental rats are shown in Figure 3b. The control group exhibited significantly lower PGE2 levels compared to both *W. coagulans* (*p* < 0.05) and the naïve group (*p* < 0.05). Similarly, the ranitidine group displayed a marked reduction in PGE2 levels relative to *W. coagulans* (*p* < 0.01) and the naïve group (*p* < 0.01). In contrast, PGE2 values were comparable among the lansoprazole, *W. coagulans*, and naïve groups.

#### Gastric nitric oxide

The nitric oxide concentration of the mucosal homogenate of the experimental rats is presented in Figure 3c. Nitric oxide values were significantly lower in *W. coagulans* (*p* < 0.01), Lansoprazole (*p* < 0.001), Ranitidine (*p* < 0.001) and Control (*p* < 0.01) groups compared with Naïve, nonetheless, these groups were comparable in NO content.

#### Oxidative stress biomarkers

**Figure 4:**
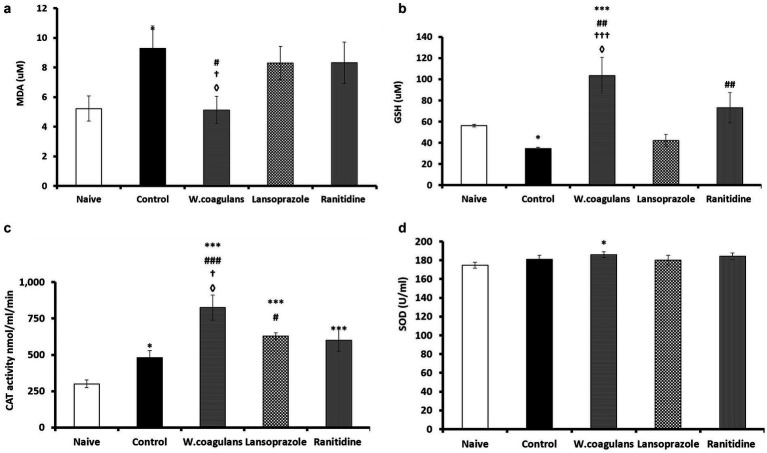
**(a)** MDA (μM) in the gastric mucosa of rats pretreated either with *W. coagulans* fruit extract (10 mg/kg); Lansoprazole (10 mg/kg); Ranitidine (20 mg/kg) or left as positive Control which were then challenged with acidified ethanol per os. Naïve was left untreated. Signs denote: *Sig. from Naïve; #Sig. from Control; †Sig from Lansoprazole; ◊Sig from Ranitidine (*p* < 0.05). **(b)** GSH (μM) in the gastric mucosa of rats pretreated either with *W. coagulans* fruit extract (10 mg/kg); Lansoprazole (10 mg/kg); Ranitidine (20 mg/kg) or left as positive Control which were then challenged with acidified ethanol per os. Naïve was left untreated. Signs denote: *Sig. from Naïve; #Sig. from Control; †Sig from Lansoprazole; ◊Sig from Ranitidine. Increasing number of signs denote increasing levels of sig. (*p* < 0.05; *p* < 0.01; *p* < 0.001). **(c)** CAT (nmol/ml/min) in the gastric mucosa of rats pretreated either with *W. coagulans* fruit extract (10 mg/kg); Lansoprazole (10 mg/kg); Ranitidine (20 mg/kg) or left as positive Control; which were then challenged with acidified ethanol per os. Naïve was left untreated. Signs denote: *Sig. from Naïve; #Sig. from Control; †Sig from Lansoprazole; ◊Sig from Ranitidine. Increasing number of signs denote increasing levels of sig. (*p* < 0.05; *p* < 0.01; *p* < 0.001). **(d)** SOD (U/ml) in the gastric mucosa of rats pretreated either with *W. coagulans* fruit extract (10 mg/kg); Lansoprazole (10 mg/kg); Ranitidine (20 mg/kg) or left as positive Control which were then challenged with acidified ethanol per os. Naïve was left untreated. Sign denotes: *Sig. from Naïve (*p* < 0.05).

Figures 4a–d present results of oxidative stress biomarkers (MDA, GSH, CAT, SOD) quantified from gastric mucosal homogenate of rats pretreated with *W. coagulans* fruit extract or antiulcer drugs and challenged with acidified ethanol as ulcerogenic agent.

#### MDA concentration in gastric homogenate

Figure 4a presents the MDA concentration in the gastric homogenate of rats across the *W. coagulans*, Lansoprazole, Ranitidine, Control, and Naïve groups. Rats treated with *W. coagulans* extract showed the lowest MDA levels, significantly lower than those in the Control, Lansoprazole, and Ranitidine groups (*p* < 0.05), and comparable to the Naïve group. The Control group exhibited a significantly higher MDA concentration than the Naïve group (*p* < 0.05). While the Lansoprazole and Ranitidine groups showed similar MDA levels to each other, both tended to have higher, though not statistically significant, MDA concentrations compared to the Naïve group.

#### GSH concentration

GSH concentration in gastric homogenate of the different groups of experimental rats is presented in Figure 4b. GSH level was significantly higher in *W. coagulans* relative to Control (*p* < 0.01), Lansoprazole (*p* < 0.001), Ranitidine (*p* < 0.05), and Naïve (*p* < 0.001) groups. The Ranitidine group exhibited significantly higher (*p* < 0.01) GSH values compared to Control. GSH level for Lansoprazole was not different from that of Control and Naïve, however Control group displayed significantly (*p* < 0.05) lower GSH values relative to Naïve.

#### Catalase activity

Catalase activity in the gastric homogenate of the experimental rats is presented in Figure 4c. CAT activity in *W. coagulans* group of rats was significantly higher than Control (*p* < 0.001), Lansoprazole (*p* < 0.05), Ranitidine (*p* < 0.05), and Naïve (*p* < 0.001) groups. In addition, CAT displayed higher values in Lansoprazole compared to Control (*p* < 0.05) and Naïve (*p* < 0.001) groups. Similarly, CAT activity was higher in Ranitidine (*p* < 0.001) and Control (*p* < 0.05) groups compared to Naïve. However, the enzyme activity did not change significantly between Ranitidine and Control.

#### Superoxide dismutase activity

SOD activity in the gastric homogenate of the experimental rats is presented in Figure 4d. *Withania coagulans* exhibited significantly (*p* < 0.05) higher SOD concentration compared to Naïve, otherwise the rest of the groups’ data were comparable.

#### Gastric inflammatory cytokine markers

**Figure 5:**
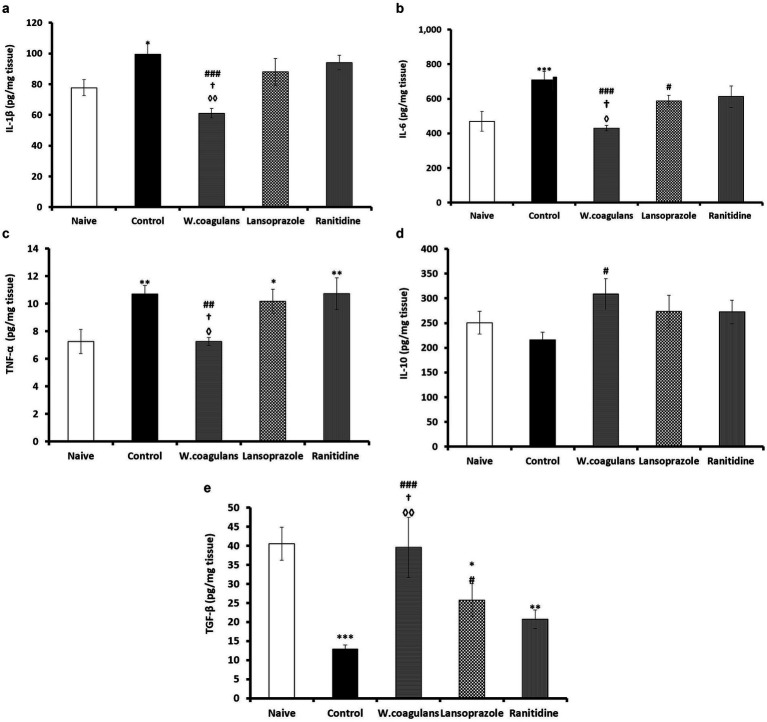
**(a)** IL-1ẞ (pg/mg tissue) in the gastric mucosa of rats pretreated either with *W. coagulans* fruit extract (10 mg/kg); Lansoprazole (10 mg/kg); Ranitidine (20 mg/kg) or left as positive Control which were then challenged with acidified ethanol per os. Naïve was left untreated. Signs denote: *Sig. from Naïve; #Sig. from Control; †Sig from Lansoprazole; ◊Sig from Ranitidine. Increasing number of signs denote increasing levels of sig. (*p* < 0.05; *p* < 0.01; *p* < 0.001). **(b)** IL-6 (pg/mg tissue) in the gastric mucosa of rats pretreated either with *W. coagulans* fruit extract (10 mg/kg); Lansoprazole (10 mg/kg); Ranitidine (20 mg/kg) or left as positive Control which were then challenged with acidified ethanol per os. Naïve was left untreated. Signs denote: *Sig. from Naïve; #Sig. from Control; †Sig. from Lansoprazole; ◊Sig. from Ranitidine. Increasing number of signs denote increasing levels of sig. (*p* < 0.05; *p* < 0.001). **(c)** TNF-a (pg/mg tissue) in the gastric mucosa of rats pretreated either with *W. coagulans* fruit extract (10 mg/kg); Lansoprazole (10 mg/kg); Ranitidine (20 mg/kg) or left as positive Control which were then challenged with acidified ethanol per os. Naïve was left untreated. Signs denote: *Sig. from Naïve; #Sig. from Control; †Sig from Lansoprazole; ◊Sig from Ranitidine. Increasing number of signs denote increasing levels of sig. (*p* < 0.05; *p* < 0.01). **(d)** IL-10 (pg/mg tissue) in the gastric mucosa of rats pretreated either with *W. coagulans* fruit extract (10 mg/kg); Lansoprazole (10 mg/kg); Ranitidine (20 mg/kg) or left as positive Control which were then challenged with acidified ethanol per os. Naïve was left untreated. Sign # denotes: Sig. from Control (*p* < 0.05). **(e)** TGF-*β* (pg/mg tissue) in the gastric mucosa of rats pretreated either with *W. Coagulans* fruit extract (10 mg/kg); Lansoprazole (10 mg/kg); Ranitidine (20 mg/kg) or left as positive Control which were then challenged with acidified ethanol per os. Naïve was left untreated. Signs denote; *Sig. from Naïve; #Sig. from Control; †Sig from Lansoprazole; ◊Sig from Ranitidine. Increasing number of signs denote increasing levels of sig. (*p* < 0.05; *p* < 0.01; *p* < 0.001).

Inflammatory/anti-inflammatory cytokines (IL-1*β*; IL-6; TNFα, IL10, TGF-β) values in gastric homogenate in experimental rats are presented in Figures 5a–e. In Figure 5a, the *W. coagulans* group showed significantly lower IL-1β levels than Control (*p* < 0.001), Lansoprazole (*p* < 0.05) and Ranitidine (*p* < 0.01) groups. The Control group displayed a significantly higher (*p* < 0.05) IL-1β levels compared to Naïve, otherwise the rest of data were not statistically different.

The *W. coagulans* group revealed the lowest IL-6 concentration in comparison with Control (*p* < 0.001), Lansoprazole (*p* < 0.05) and Ranitidine (*p* < 0.05) groups (Figure 5b). The Lansoprazole group showed significantly lower IL-6 compared to Control (*p* < 0.05) while the Control exhibited significantly higher IL- 6 value compared to Naïve (*p* < 0.001), whereas other IL-6 data were comparable between groups.

The *W. coagulans* group was lowest in TNFα concentration compared with Control (*p* < 0.01), Lansoprazole (*p* < 0.05) and Ranitidine (*p* < 0.05) groups (Figure 5c). On the other hand, control (*p* < 0.01), Lansoprazole (*p* < 0.05) and Ranitidine (*p* < 0.01) groups manifested significantly higher TNFα concentrations in comparison with the Naïve, but attained comparable values.

The *W. coagulans* group displayed significantly higher (*p* < 0.05) IL-10 concentration compared to Control while other IL-10 data were comparable between groups.

The concentration of TGF-*β* in mucosal homogenate of the experimental rats is presented in Figure 5e. *W. coagulans* exhibited the highest TGF-β levels compared to Control (*p* < 0.001), Lansoprazole (*p* < 0.05) and Ranitidine (*p* < 0.01) groups but comparable to Naïve. Lansoprazole group attained significantly higher (*p* < 0.05) levels than that of Control. However, Lansoprazole (*p* < 0.05), Ranitidine (*p* < 0.01) and Control (*p* < 0.001) groups exhibited significantly lower TGF-β levels compared to Naïve.

#### Gastric myeloperoxidase activity

**Figure 6:**
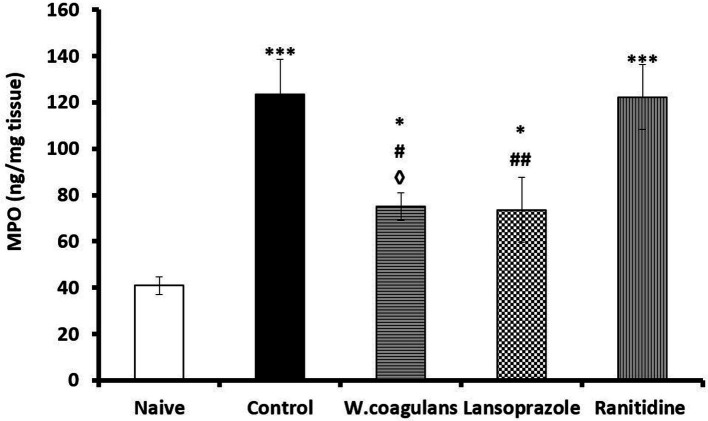
MPO (ng/mg tissue) in the gastric mucosa of rats pretreated either with *W. coagulans* fruit extract (10 mg/kg); Lansoprazole (10 mg/kg); Ranitidine (20 mg/kg) or left as positive Control which were then challenged with acidified ethanol per os. Naïve was left untreated. Signs denote: *Sig. from Naïve; #Sig. from Control; ◊Sig from Ranitidine. Increasing number of signs denote increasing levels of sig. (*p* < 0.05; *p* < 0.01; *p* < 0.001).

The myeloperoxidase (MPO) activities in the gastric homogenate of *W. coagulans*, Lansoprazole, Ranitidine, Control and Naïve are presented in Figure 6. The MPO activity in *W. coagulans* was significantly lower (*p* < 0.05) than that of Control and Ranitidine groups but comparable to that of the Lansoprazole group. On the other hand, MPO activity of *W. coagulans* (*p* < 0.05), Control (*p* < 0.001), Lansoprazole (*p* < 0.05) and Ranitidine (*p* < 0.001) groups were significantly higher than that reported for Naïve. Lansoprazole displayed significantly lower (*p* < 0.01) MPO concentration compared to Control.

#### Histopathology results

**Figure 7:**
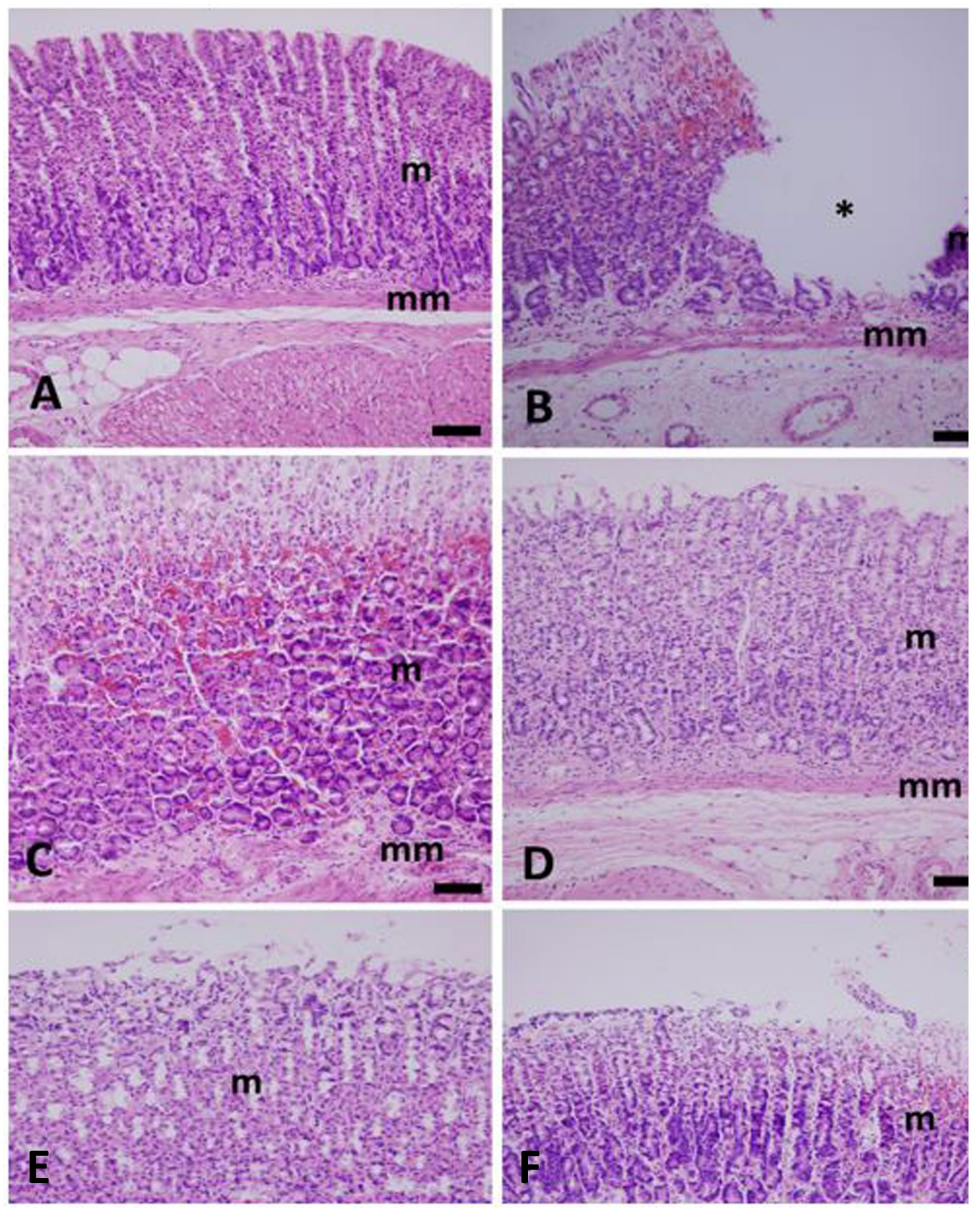
Light micrographs of hematoxylin–eosin-(H & E) stained sections of stomach of untreated and those treated with either Withania, Lansoprazole or Ranitidine. **(A)** Naive rats with normal gastric mucosa with pits, gastric glands, muscularis mucosa and submucosa; **(B)** Positive control with ulcer crater and hemorrhage. **(C)** Positive control group, as in B, showing severe hemorrhage and degeneration in the mucosal layer of the stomach. **(D)** Withania coagulans-treated group showing apical sloughing of gastric epithelium. No ulcer was observed; **(E)** Lansoprazole-treated group displaying apical sloughing and vacuolar degeneration of the mucosa; **(F)** Ranitidine-treated group showing focal hemorrhage, epithelial necrosis but no ulcer. m, mucosa; mm, muscularis mucosae; *ulcer. Scale bar = 50 μm.

All experimental rats receiving *Withania* extract, Lansoprazole and Ranitidine treatments, did not develop gastric ulcer and the lesions observed were mild but with slight variation between treatments. In the *Withania* group (Figure 7D), the gastric lesions were milder compared to other treatment groups and comprising focal epithelial erosion and/or degeneration epically, focal hemorrhage and infiltration of polymorphonuclear leukocytes. The gastric lesions in the Lansoprazole group (Figure 7E) included epithelial erosion, glandular dilatation and cellular degeneration and/or necrosis but no hemorrhage was observed. The Ranitidine group (Figure 7F) displayed more marked lesions than the *Withania* and lansoprazole groups comprising focal epithelial erosion epically, cellular degeneration and/or necrosis, focal hemorrhage and infiltration of polymorphonuclear leukocytes. On the other hand, the Control group developed acute inflammation with ulcer formation (Figure 7B), epithelial erosion with necrotic debris, focal loss of gastric glands, cellular necrosis and/or degeneration and severe hemorrhage (Figure 7C) though the entire mucosa with infiltration of polymorphonuclear cells. No lesion was observed in the Naïve group (Figure 7A) where the mucosa, submucosa and muscularis externa layers were intact and devoid of epithelial erosions and leukocytic infiltration.

## Discussion

The popularity of herbal medicine for treatment of PUD has increased, as they are natural, cheap, effective and with no or little side effects if properly administered. Ulcer drugs such as Lansoprazole and Ranitidine are effectively used in the treatment of PUD. However, they are not without side effects which can encourage some patients to look for alternative medicine (35, 56). *Withania coagulans* has been successfully used in the treatment of diabetes and wound healing beside other benefits (45–47). In this work, we further investigated the antiulcerogenic potential of *W. coagulans* fruit extract. From a dose response curve 10 mg/kg fruit extract has been suggested as the optimum effective dose against ethanol-induced gastric ulcer in rats. *Withania coagulans* fruit extract administered at 10 mg/kg was shown to be equally effective in gastric ulcer protection as Ranitidine (20 mg/kg). However, Lansoprazole (10 mg/kg), attained the minimum ulcer index.

Gastric acid secretion is intricately regulated by a network of signaling molecules, including gastrin, histamine, somatostatin, acetylcholine, and various neuropeptides. Gastrin plays a key role by stimulating enterochromaffin-like (ECL) cells in the stomach to release histamine. This histamine then binds to H2 receptors on parietal cells, triggering acid secretion. Activation of these receptors increases levels of cyclic adenosine monophosphate (cAMP), which in turn activates protein kinase A (PKA). PKA then phosphorylates specific proteins involved in hydrogen ion transport, ultimately enhancing hydrochloric acid (HCl) production.

H2 receptor antagonists, or H2 blockers, inhibit this pathway by preventing histamine from binding to its receptors, thereby reducing gastric acid secretion (57).

Conversely, somatostatin inhibits acid secretion by binding to somatostatin receptor 2 (SSTR2) on parietal cells, ECL cells, and gastric neurons. Interestingly, extract from *W. coagulans* mimics the action of H2 blockers. In this study, rats treated with Withania extract showed the lowest levels of gastric acid secretion and the highest somatostatin concentrations in gastric juice following stomach ligation. This suggests that *W. coagulans* fruit extract exerts its gastroprotective effects by blocking H2 receptors and stimulating delta (D) cells in the stomach to produce somatostatin. The increased somatostatin then activates SSTR2 receptors, thereby suppressing histamine release and further acid production.

In contrast, administration of Dimaprit, an exogenous histamine receptors (H2R) agonist (58, 59), alone significantly stimulated H2R in parietal cells with increased gastric acid output and minimal somatostatin release from D cells. However, pretreatment of rats with *Withania* fruit extract and challenge with a concomitant dose of Dimaprit, modulated both gastric acid output and somatostatin secretion to attain intermediate values compared to groups given either the extract or Dimaprit alone. *Withania* fruit extract can reduce excessive gastric acid in a dose dependent manner by inhibition of histamine and stimulation of somatostatin release in a similar mechanism as Ranitidine and the medicinal plant *Cudrania tricuspidata* which exerts a direct H2R antagonistic effects (59).

Malondialdehyde is a product of lipid peroxidation and an oxidative stress biomarker (60). Administration of *W. coagulans fruit* extract to rats followed by a subsequent dose of acidified ethanol, induced protective effects against ROS by illustrating minimal MDA concentration, comparable only to Naïve whereas Lansoprazole, Ranitidine and Control groups given similar acidified-alcohol regime, significantly provoked tissue MDA. The protective effect of *Withania* extract against ROS species is apt to be conferred by the higher GSH and CAT levels (61), which is in line with previous reports on the antioxidant properties of *Withania* derived from *in-vivo* and *in-vitro* systems (45, 46, 51). The plant fruit is rich in Withaferin A which activates Nrf2 transcription factor to induce endogenous antioxidants [GSH, SOD1, SOD2, CAT, GPx4 and heme oxygenase 1 (HO-1)] (62, 63). GSH, mainly in the cytosol, acts directly on or is pumped into the mitochondria to protect cellular organelles from both endogenous and exogenous reactive oxygen or nitrogen species (64). SOD2, a mitochondrial enzyme that converts ROS [superoxide radical anion (O2-) into hydrogen peroxide thus protecting Krebs enzymes from damage] (65). This with hydrogen peroxide is converted by CAT (from peroxisomes) into water and oxygen. Moreover, the *W. coagulans* fruit is rich in ascorbic acid which is well known as a ROS scavenger (66).

Myeloperoxidase is a lysosomal enzyme, abundantly stored in the neutrophil’s granules and in macrophages and it is clinically used as neutrophil inflammatory biomarker (67). MPO is released extracellularly during acute inflammation and oxidative stress to catalyze production of hypochlorous acid (HOCl) and tyrosyl radical which are cytotoxic agents encountered in tissue oxidative injury (68). All the experimental group of rats showed elevated MPO activity compared with Naïve, which was in line with the acute inflammation currently induced by acid with infiltration of polymorphonuclear leukocytes observed morphologically in the gastric epithelium of these groups. MPO level was significantly attenuated in *W. coagulans* and Lansoprazole compared to Control and Ranitidine. However, due to the short experimental period further observation is required before drawing any conclusions that *Withania* is effective in reducing MPO and neutrophil activities like that observed in ingredients of some essential oils (69).

Interleukins IL-1β, TNF*α* and IL-6 are proinflammatory markers produced by many cell types including activated macrophages/monocytes and activated subsets of T lymphocytes and their levels increase significantly in inflammatory conditions (17, 70). These interleukins complex with receptors upon activation by noxious agents to stimulate different signaling transduction cascades to produce cytokine mediated inflammatory responses (17, 71). The level of IL-1β, IL-6 and TNF-*α* remained unchanged in the *Withania group* of rats compared to Naïve which affirmed that the extract conferred some protective effects upon gastric mucosa where only focal degenerative changes and rarely focal hemorrhage were observed. On the other hand, the Control group exhibited the highest concentrations of these cytokines with an inevitable tissue necrosis, sloughing and ulcer formation accompanied with severe hemorrhage and leukocytic infiltration. The Lansoprazole and Ranitidine groups depicted increased concentrations of IL-1*β*, IL-6 and TNF-α with less severe focal cellular degeneration and/or necrosis and/or hemorrhage (mainly seen in Ranitidine group but not Lansoprazole) compared to Control but more pronounced than lesions observed in the *Withania* group. This may point to the superiority of *Withania* fruit extract to these drugs in this respect. It has been reported that *Withania* is rich in the compounds Withaferin A and 3- *β*-hydroxy- 2, 3- dihydrwithanolide F, which possess anti-inflammatory and hepatoprotective activities (45, 51, 72). Anti-inflammatory cytokines are required to balance excessive pro-inflammatory cytokines production otherwise, any disparity may exacerbate the inflammatory progression (30). IL-10 is a key anti- inflammatory cytokine secreted by activated monocytes and Th2 lymphocyte cells subset shortly after a pro-inflammatory response to culminate tissue damage but with tightly regulated production (73). IL-10 and IL-6 share a common JAK/STAT3 transcription factor pathway to induce a signaling effect however, SOCS3 regulates their diversity by selectively inhibiting lL-6 signaling but not IL-10 (74). Upon stimulation, IL-10 complexes with receptors emitting signaling cascades to down regulate synthesis of TNFα, IL-1 *β*, IL-6 and other cytokines (75, 76). The significant elevation of IL-10 in *Withania* fruit extract group compared with Control, corroborated by a significantly lower pro-inflammatory cytokines TNFα, IL- 1 *β*, IL-6 and less marked degenerative gastric lesions have been clear evidence that the *W. coagulans* possesses anti-inflammatory and antiulcerogenic effects. TGF-*β* is secreted by many cells, activated by proteases to interact with receptors and consequently generate signaling cascades which sometime may have conflicting effects. TGF-β regulates proliferation of different cell types, maintains cell integrity, reduces inflammation and improves gastric ulcer healing by fibroblast activation among other functions (29, 77, 78). In this regard, TGF-β suppresses cytokine production by inhibiting macrophage (M1) and T helper cell 1 (Th1) activities by counteracting IL-1β, IL-2, IL-6, and TNFα and inducing IL-1ra (79). On the other hand, the suppression of TGF-β secretion intensifies the degree of inflammation especially with H pylori ulcer lesions (80, 81). The comparable levels of TGF-β in the *Withania* group and that of Naïve and the significantly lower TGF-β levels in the Control, Lansoprazole and Ranitidine groups implies that *Withania* fruit extract possesses an anti-inflammatory effect against ethanol-induced gastric ulcer and may possibly be superior to Lansoprazole and Ranitidine in efficacy.

Some medicinal plants elicit their protective effects to gastric mucosa from acid and pepsin by stimulation of mucus secretion (1, 82). The mucus produced in gastric mucosa of the *Withania* group was like that produced by the Naïve group which might indicate to have protective effect. However, it remains to be seen if the *W. coagulans* fruit extract has a direct effect on mucus production as some other plants (1, 82). The mucus production in the gastric mucosa of Lansoprazole, Ranitidine and Control groups was even much reduced compared to *Withania* and Naïve groups.

PGE2, a prostaglandin produced by COX from arachidonic acid, is abundant in gastric mucosa and increases gastric mucus synthesis and viscosity, bicarbonate production, inhibits histamine release/acid secretion and ultimately contributes to protection against gastric ulcers (83–85). It has also been shown that PGE2 safeguards against damage of gastric and duodenum mucosa independent of its inhibitory effect on gastric acid production (86). The high PGE2 level in the *W. coagulans* fruit extract group might have a protective effect as it was significantly higher than the ranitidine and control group and was similar to the lansoprazole and naïve groups. The PGE2 level in the *W. coagulans* group might have a protective effect. NO and prostaglandins syntheses were observed to occur concurrently in tissues but the mechanism through which the enzymes, NOs synthase and COX, collate in synthesizing NO and prostaglandins these products remained elusive (87, 88). NO mediates relaxation of arteriolar smooth muscles, increases blood flow, augments new blood vessel formation, reacts directly with superoxide anion to reduce oxidative stress and modulates inflammatory reactions etc. (89, 90). As NO levels were significantly reduced in all treatment groups compared to Naïve this may indicate either reduced synthesis or excessive usage of NO for ROS scavenging or both.

## Conclusion

The present study demonstrates that *W. coagulans* fruit extract at a dose of 10 mg/kg body weight confers significant protection against alcohol-induced gastric ulcers in rats. Its efficacy is comparable to, or exceeds, that of standard anti-ulcer drugs such as Lansoprazole (10 mg/kg) and Ranitidine (20 mg/kg). The gastroprotective effects of the extract are mediated, in part, by the stimulation of somatostatin secretion, which leads to reduced histamine release and subsequent suppression of gastric acid secretion. Furthermore, *W. coagulans* exhibits notable anti-inflammatory and antioxidant activities by modulating cytokine profiles and enhancing the activity of key endogenous antioxidants including glutathione (GSH), superoxide dismutase (SOD), and catalase (CAT), thereby mitigating oxidative stress. Additional protective mechanisms may involve increased mucus production and prostaglandin E2 (PGE2) synthesis, while nitric oxide (NO) appears to play a minimal role. These findings suggest that *W. coagulans* fruit extract holds promise as a potential therapeutic agent for peptic ulcer disease. However, further clinical investigations are required to confirm its efficacy in humans, establish appropriate therapeutic dosing, and evaluate its safety profile.

## Strengths and limitations of the study

A key strength of this study is the demonstration that Withania fruit extract offers a promising protective effect against gastric ulcers at a relatively low dose (10 mg/kg). The extract exerts its therapeutic effects by inhibiting histamine production and promoting antioxidant and anti-inflammatory activities. Additionally, Withania fruits are non-toxic and commonly used in the preparation of cottage cheese. They are naturally abundant and widely cultivated, making them an accessible and safe option for therapeutic use. However, the study has certain limitations. A comprehensive analysis of the plant’s active constituents particularly vitamins A, C, and E, which are natural free radical scavengers was not performed. Similarly, phenolic and flavonoid content, which contribute to antioxidant and anti-inflammatory effects, were not fully analyzed in the context of this study, although some phytochemical evaluations have been conducted previously for other purposes. Moreover, important inflammatory markers such as interleukins IL-4 and IL-12 were not assessed.

## Data Availability

The raw data supporting the conclusions of this article will be made available by the authors, without undue reservation.
